# Does Invisalign Outperform Fixed Appliance in Treating Vertical Discrepancies?

**DOI:** 10.7759/cureus.65973

**Published:** 2024-08-01

**Authors:** Ghadir Mansour Alawdi, Meeral Fahad Al Fahad, Shaden Bander Al Muzher, Aiysha Huseen Alfaifi, Aysha Mohamed Hazeem, Rawan Sulaiman Dakheel, Roaa Hassan Jan, Laith Maher Al-Qutub, Lamia Hajjaj Alharbi, Ahmed Khalil

**Affiliations:** 1 Dentistry, Batterjee Medical College, Jeddah, SAU; 2 Dentistry, King Khalid University, Abha, SAU; 3 Dentistry, Private Practice, Dammam, SAU; 4 Dentistry, Private Practice, Al-Madinah, SAU; 5 Dentistry, Prince Mansour Military Hospital, Taif, SAU; 6 Dentistry, Riyadh Elm University, Riyadh, SAU; 7 Dentistry, University of Hail, Hail, SAU; 8 Orthodontics, Private Practice, Alexandria, EGY

**Keywords:** clear aligner therapy (cat), anterior open bite (aob), vertical, malocclusion, invisalign

## Abstract

Aligners became popular among adult patients for their superior aesthetics and comfort in comparison to conventional fixed appliances. It has undergone numerous enhancements over time, allowing it to address more complex malocclusions. Many researchers argued that managing vertical discrepancies is more challenging than addressing anteroposterior issues. This complexity arose from the mechanical requirements for treatment and the required mechanics to prevent relapse. Studies assessing the treatment outcome of anterior open bite closure using clear aligners have yielded conflicting results regarding the mechanisms of bite closure. Proposed mechanisms included extrusion of upper or lower incisors, lingual tipping of upper or lower incisors, intrusion of upper or lower molars, counterclockwise rotation of the mandible, or various combinations of these mechanisms. The research highlighted the biomechanical challenges associated with using aligners for the treatment of deep bites as mandibular incisor intrusion and leveling the curve of Spee remain among the least predictable movements. Given the widespread use of aligners, it is imperative to rigorously assess the effectiveness of clear aligners in achieving overbite correction to ensure they deliver the desired outcome. This review aimed to assess the performance of Invisalign in the management of vertical discrepancies. It sought to identify the dentoskeletal effects of clear aligners in addressing deep bite and anterior open bite cases, understand the mechanisms behind overbite correction, and provide a comprehensive overview of the existing research on this topic.

## Introduction and background

Since Align Technology introduced Invisalign approximately two decades ago, it has gained popularity among adult patients due to its superior aesthetics and comfort compared to traditional fixed orthodontic appliances [1,2]. It was originally marketed as an aesthetic alternative for simple cases without skeletal discrepancies, primarily involving mild-to-moderate crowding cases [3]. However, the Invisalign system has undergone numerous enhancements over time to improve alignment and occlusion capabilities, allowing it to address more complex malocclusions.

Many researchers argued that managing vertical discrepancies is more challenging than addressing anteroposterior issues [4]. This complexity stemmed from the mechanical requirements for treatment and the required mechanics to prevent relapse [5]. Anterior open bite presented a significant challenge in orthodontics due to its complex etiologies and association with various malocclusions [6-8]. Treatment of anterior open bite is often pursued not only for aesthetic reasons but also for functional concerns. These functional issues include speech and mastication difficulties along with the risk of buccal segment wear due to lack of occlusion of anterior teeth [9]. Additionally, vertical control has consistently been a significant challenge in orthodontic treatment [10,11]. Different treatment modalities have been suggested for addressing anterior open bite, including the use of mini-screws or mini-plates for intrusion of buccal segment [12-17], fixed orthodontic appliance combined with anterior intermaxillary elastics [18], posterior bite plane [19,20], multiloop edgewise archwire mechanics [21-23], and orthognathic surgery via LeFort I maxillary impaction [24,25]. The treatment of anterior open bite using clear aligners has shown conflicting results regarding the mechanism of bite closure. Proposed mechanisms for correction included extrusion of upper or lower incisors, lingual tipping of upper or lower incisors, intrusion of upper or lower molars, counterclockwise rotation of the mandible, or various combinations of these mechanisms [26]. Aligners were found to work similarly to occlusal bite-blocks used in fixed appliances due to their coverage of the posterior teeth [27]. This coverage helped to prevent the extrusion of buccal segments or even promote their intrusion [28]. However, this hypothesis remains unverified. Studies focused on vertical discrepancies have centered on deep bite correction using aligners. In opposition, research highlighted the biomechanical challenges associated with the use of aligners for mandibular incisor intrusion and leveling the curve of Spee, which remain among the least predictable movements [29,30]. This led to an extended treatment duration and minimal improvement in overbite, despite the utilization of precision bite ramps and optimized attachments [31].

Given the widespread use of aligners, it is imperative to rigorously assess the effectiveness of Invisalign in achieving overbite correction to ensure they deliver the desired outcome. This narrative review aimed to assess the efficiency of Invisalign in the management of deep bite and anterior open bite cases. It sought to identify the dentoskeletal effects of clear aligners in addressing vertical discrepancies, understand the mechanisms behind overbite correction, and provide a comprehensive overview of the existing research on this topic.

## Review

Search strategy

A comprehensive literature search was conducted across several reputable electronic databases, including PubMed, Embase, MEDLINE, and Google Scholar, to identify peer-reviewed articles relevant to the review up to February 1, 2024. The inclusion criteria encompassed original research articles, review articles, systematic reviews, clinical trials, and animal studies, all published in English. The screening process began with an initial evaluation of titles and abstracts to assess their relevance. Articles meeting the initial criteria were then subjected to a full-text review to confirm their relevance to the review topic. Excluded from the search were non-English publications, studies focusing on unrelated aspects of orthodontic tooth movement, opinion pieces, editorials, and case reports with limited applicability.

The evolution of invisible appliances

The main advantage of aligners over traditional metal braces is their invisible appearance and greater comfort [32]. Initially, clear aligners were developed for the final stages of orthodontic treatment or to correct minor malalignment of teeth [33]. However, advancement in clear aligner therapy enabled their use in treating moderate to severe malocclusions. Various thermoplastic materials such as polyvinyl chloride, polyurethane, polyethylene terephthalate, and polyethylene terephthalate glycol have been utilized for thermoformed aligners [34]. The design process begins with virtual planning software, using plaster impressions or direct digital 3D intraoral scan. Each aligner in the treatment set requires a 3D model created using 3D printing, stereolithography, or material jetting [35]. The aligner trays are then fabricated by molding the clear material over the 3D model through vacuum forming, followed by trimming and polishing the margins. Yet, the lengthy process along with the multiple intermediate steps remain significant drawbacks [36]. Research found that the thermoforming process reduces the thickness of the aligners compared to the original thickness [37]. The mechanical properties of the thermoplastic material are crucial for achieving desired clinical outcomes, especially for complex orthodontic movements [38]. Thermoformed aligners can vary in thickness from anterior to posterior teeth which was found to impact their performance in achieving the desired movement. Not to mention the burden on the environment [39]. The limitations of conventional vacuum thermoforming have driven the development of direct 3D-printed aligners. This era was found to eliminate errors associated with intermediate steps, allow for dimensionally controlled differential thickness tailored to individual cases, and result in better force application [40]. However, the biocompatibility of 3D-printed aligners has not yet been thoroughly evaluated.

Correction of deep overbite malocclusion

A series of studies have assessed the efficiency and predictability of clear aligners in correcting deep bites. Muro et al. conducted a scoping review that found clear aligners effective for mild-to-moderate crowding, but less predictable for overbite correction, with buccolingual tipping being the most reliable movement [41]. Similarly, Shahabuddin et al. performed a retrospective study on 24 patients, analyzing their initial, predicted, and achieved models [42]. They found an average overbite correction of only 33%, necessitating overcorrection and additional refinement treatments. In the same line, Kang et al. retrospectively analyzed 20 deep bite patients and reported significant discrepancies between predicted and achieved outcomes, with an initial mean accuracy of 37.63% dropping to 11.19% after multiple refinements [43]. Conversely, a study evaluated 120 adult patients and found a median overbite reduction of 1.5 mm primarily due to incisor repositioning, though with minimal changes in molar vertical position and mandibular plane angle [44]. This study concluded that Invisalign effectively manages the vertical dimension of overbites. Henick et al. compared the skeletal and dentoalveolar effects of Invisalign's G5 protocol with fixed appliances in 48 adults with skeletal deep bites [45]. Both groups showed significant improvements in the overbite depth indicator, though fixed appliances resulted in more pronounced skeletal changes. Similarly, Galan-Lopez et al. systematically reviewed the efficiency of Invisalign, concluding that fixed appliances offer superior accuracy, especially for vertical movements and complex cases [46]. Fujiyama et al. compared Invisalign and fixed appliances in 50 patients with severe deep overbites, finding significant improvements in both groups, with Invisalign showing notable vertical changes [47]. Rozzi et al. evaluated the leveling of the curve of Spee in two groups, 30 patients treated with Invisalign and 32 with fixed appliances [48]. Cephalometric analysis showed significant leveling in both groups, though Invisalign provided better control over incisor proclination. Conversely, Kravitz et al. discussed the challenges of deep bite correction with aligners, highlighting the need for strategies like virtual case setups, attachment designs, elastics, and bite ramps to improve clinical success [49]. A retrospective study by Blundell et al. compared different aligner materials and found that neither SmartTrack with precision bite ramps nor EX30 material significantly improved predictability, achieving 43.4% and 55.1% of the prescribed reduction, respectively [50]. This indicates limited effectiveness in achieving desired overbite outcomes with these modifications.

**Figure 1:**
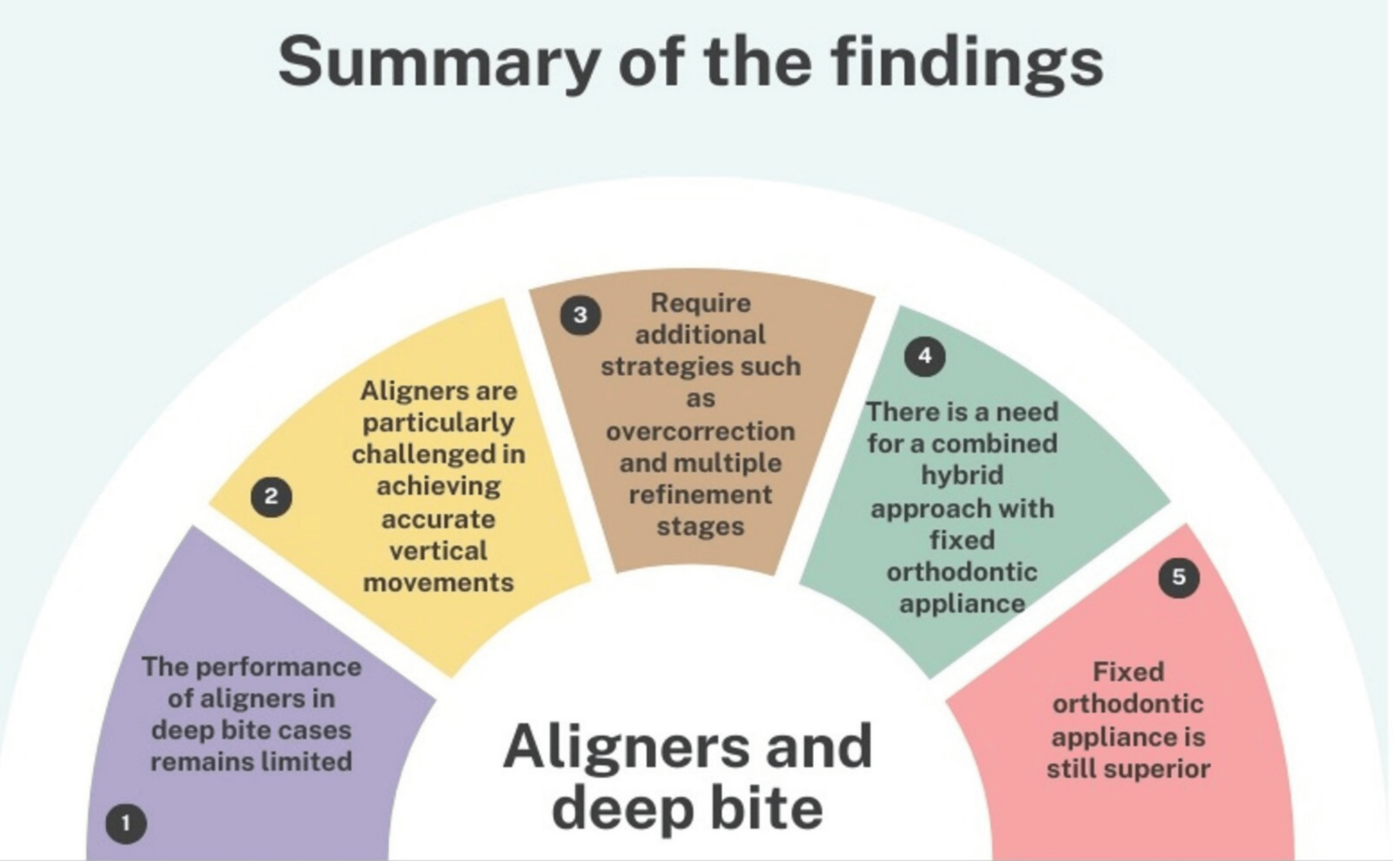
Summary of the findings with regard to the performance of aligners in deep bite cases. The image is created by the author (AK) of this study with the help of www.canva.com.

The predictability and effectiveness of clear aligners in deep bite cases remain limited. Aligners are particularly challenged in achieving accurate vertical movements and require additional strategies such as overcorrection and multiple refinement stages. Despite advancements, fixed appliances continue to provide superior control and accuracy in managing complex malocclusions [51-53]. This indicates the need for a combined hybrid approach with fixed orthodontic appliances or mini-screws to optimize treatment outcomes with clear aligners [54-58]. An overview of the included studies that assessed the efficiency of clear aligners in the management of deep bite cases is depicted in Table 1. A summary of the findings is depicted in Figure 1.

**Table 1:** An overview of the included studies that assessed the efficiency of clear aligners in the management of deep bite cases.

Studies	Study design and sample	Study objectives	Main findings
Muro et al. 2023 [41]	Review	To evaluate the effectiveness and predictability of clear aligners in overbite correction.	Clear aligners were effective for mild-to-moderate crowding but had limited predictability in overbite correction. Buccolingual tipping is the most predictable movement, while rotation, intrusion, and extrusion are less reliable.
Shahabuddin et al. 2023 [42]	Retrospective study with 24 patients	To assess the effectiveness of clear aligners in overbite correction.	Clear aligners corrected overbite by an average of 33%, with a 1.15 mm improvement after the first set of aligners. Significant discrepancies were observed between planned and achieved vertical movements and inclination changes.
Kang et al. 2024 [43]	Retrospective study with 20 patients	To evaluate the accuracy of overbite correction with clear aligners over time.	The initial mean accuracy of overbite correction was 37.63%, decreasing to 11.19% after multiple refinements. Significant discrepancies were observed between predicted and achieved outcomes.
Khosravi et al. 2017 [44]	Retrospective study with 120 patients	Assess the efficiency of clear aligners in treating overbite malocclusions.	Clear aligners reduced overbite by a median of 1.5 mm in deep bite cases through incisor repositioning, with minimal changes in molar vertical position and mandibular plane angle. Improved overbite in open bite cases through incisor extrusion.
Henick et al. 2021 [45]	Retrospective study with 48 patients	To compare the effectiveness of Invisalign's G5 protocol and fixed orthodontic appliance in improving deep bites.	Both methods showed significant improvements in overbite depth indicator and other measurements, with fixed orthodontic appliance treatment resulting in more pronounced skeletal changes. Both methods improved deep bites at skeletal and dentoalveolar levels.
Galan-Lopez et al. 2019 [46]	Systematic review	To assess the effectiveness of clear aligners in treating malocclusions.	Clear aligners can effectively treat malocclusions but are generally less precise compared to fixed appliances, facing challenges with vertical movements and complex malocclusions.
Fujiyama et al. 2022 [47]	Retrospective study with 50 patients	To compare the effectiveness of Invisalign and fixed appliances in treating severe deep overbites.	Both Invisalign and fixed appliances showed significant improvements. Notable vertical changes were observed with Invisalign, and peer assessment ratings and treatment duration were similar.
Rozzi et al. 2022 [48]	Retrospective study with 62 patients	To compare the effectiveness of Invisalign and fixed appliances in leveling the curve of Spee.	Both methods effectively leveled the curve of Spee. Invisalign provided better control over incisor proclination during intrusion, while fixed appliances showed significant posterior teeth extrusion.
Kravitz et al. 2020 [49]	Review	To explore the challenges and strategies for achieving mandibular incisor intrusion with aligners.	Achieving mandibular incisor intrusion with aligners is challenging due to factors like patient compliance, case setup, and aligner retention. Effective strategies include virtual case setups, specific attachment designs, elastics, and bite ramps.
Blundell et al. 2022 [50]	Retrospective study with 68 patients	To compare the predictability of different aligner materials in overbite reduction.	Neither SmartTrack with precision bite ramps nor EX30 material significantly improved predictability, achieving 43.4% and 55.1% of the prescribed reduction, respectively.

Anterior open bite closure

The treatment of anterior open bites presented a significant challenge in orthodontics due to the complex interplay of dental and skeletal factors involved [8]. Traditionally, fixed appliances have been the mainstay of treatment for such malocclusions, often requiring the use of auxiliaries such as mini-screws, occlusal bite blocks, and extractions to achieve desired outcomes. In recent years, clear aligners have emerged as a popular alternative to traditional fixed appliances, offering aesthetic and comfort advantages. The effectiveness of clear aligners in achieving significant anterior open bite closure was highlighted in several studies. Blundell et al. reported that approximately 66.2% of the programmed bite closure was achieved, with better outcomes when aligners were changed every two weeks [59]. Similarly, Moshiri et al. found that clear aligners effectively managed vertical dimensions through counterclockwise rotation of the mandibular plane, lower molar intrusion, and lower incisor extrusion [60]. The comparison between clear aligners and fixed appliances was further elucidated by Garnett et al. and Rask et al., who concluded that clear aligners are as effective as fixed appliances in managing vertical control and overbite correction [61,62]. In essence, Garnett et al. noted that while fixed appliances often required auxiliaries like TADs and extractions, clear aligners provided vertical control through posterior coverage of all teeth [61]. Rask et al. also found that clear aligners caused less low molar extrusion and mild mandibular backward rotation compared to fixed appliances, reinforcing their effectiveness. Harris et al. reported that clear aligners achieved bite closure through a combination of upper and lower incisor extrusion and molar intrusion, leading to mandibular autorotation [63]. This combination was effective in reducing the anterior open bite while maintaining vertical control. Suh et al. expanded on this by demonstrating the adaptability of clear aligners across different malocclusion classes [64]. The authors found that Class II patients experienced greater maxillary molar intrusion and mandibular plane angle reduction, while Class III patients showed significant mandibular incisor extrusion. On the other hand, Steele et al. offered a comparative perspective on the dentoskeletal effects of clear aligners versus miniplate-supported posterior intrusion combined with fixed appliances [65]. Their findings indicated that while miniplate-supported intrusion induced greater maxillary molar intrusion and reduced anterior face height and mandibular plane angle, clear aligners achieved bite closure primarily through incisor extrusion. Both treatment modalities effectively improved overbite.

**Figure 2:**
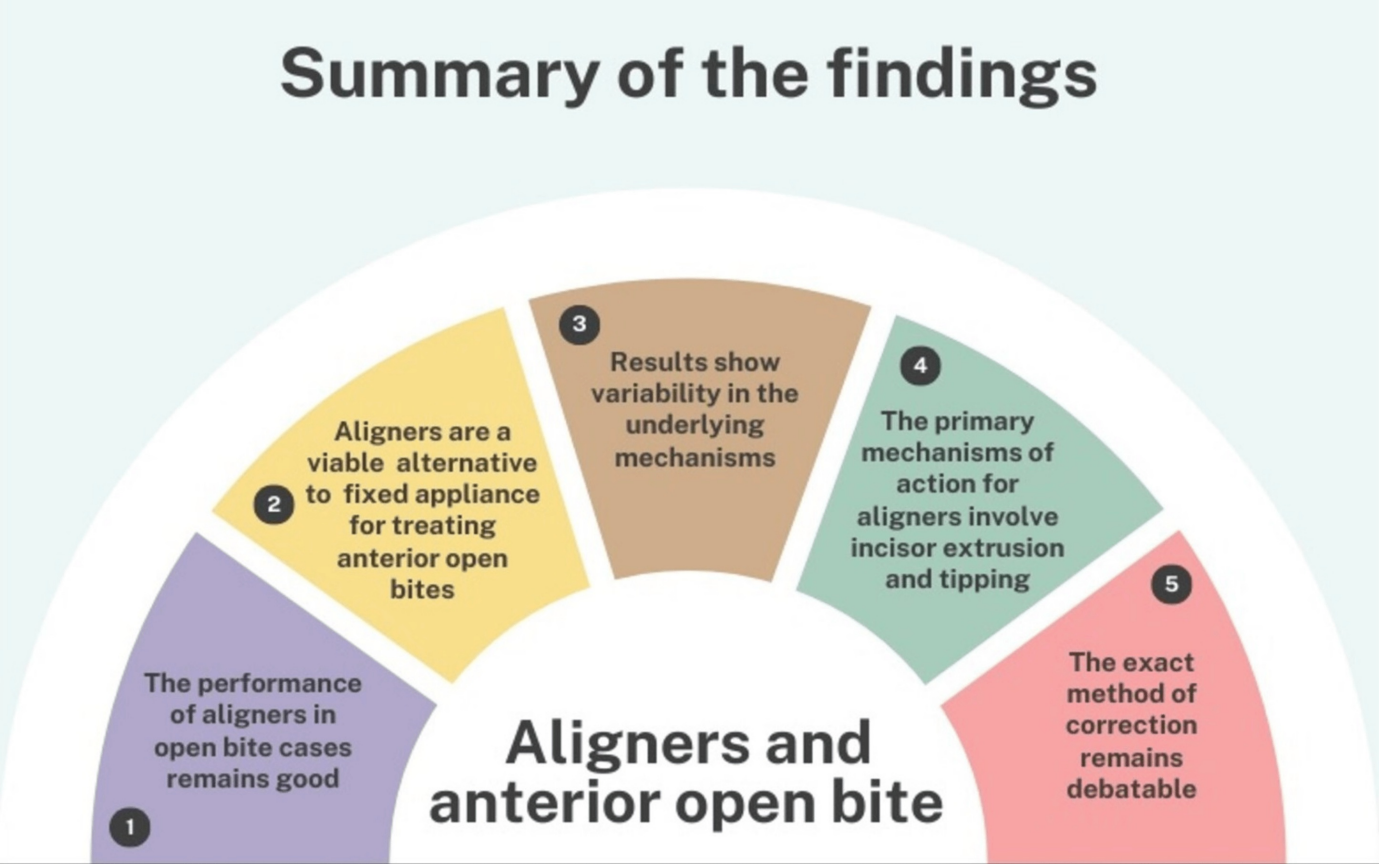
Summary of the findings with regard to the performance of aligners in anterior open bite cases. The image is created by the author (AK) of this study with the help of www.canva.com.

These studies collectively demonstrate that clear aligners are a viable and effective alternative to traditional fixed appliances for treating anterior open bites. The primary mechanisms of action for clear aligners involve incisor extrusion and tipping. Yet, results show variability in the underlying mechanisms with the exact method of correction remains debatable. A summary of the findings is depicted in Figure 2.

Deep bite versus anterior open bite cases (a comparative assessment)

Limited studies conducted a combined comparative analysis on the same study of the effectiveness of clear aligners in managing deep bite and anterior open bite cases. Khosravi et al. investigated the effectiveness of clear aligners in addressing overbites among adult patients [44]. They observed that clear aligners produced a median reduction of 1.5 mm in overbite for deep bite cases, primarily through the repositioning of incisors. Additionally, in anterior open bite cases, overbite improvement was achieved mainly through incisor extrusion. This highlights the versatility of clear aligners in addressing different types of overbites, with a particular emphasis on correcting the position of incisors. Building upon this, Talens-Cogollos et al. delved deeper into the effects of clear aligner therapy on molar intrusion, a crucial aspect in managing overbite cases [66]. Their study, which included adult patients treated with Invisalign, revealed that a significant proportion (74.2%) experienced molar intrusion, with the mandibular molars being more affected than the maxillary molars. They found an average intrusion of 0.98 mm in maxillary molars and 0.84 mm in mandibular molars. Moreover, correlations were identified between molar intrusion and cephalometric variables such as the mandibular plane angle and facial axis. These findings highlight the multifaceted nature of clear aligner therapy in overbite correction. Additionally, it's crucial for clinicians to consider factors such as patient age, treatment duration, and the degree of case complexity to optimize outcomes and tailor treatment plans accordingly.

Root resorption (a challenge in the management of vertical discrepancies)

Clear aligners are generally believed to pose a lower risk of severe root resorption compared to conventional fixed appliances [67]. Yet, conclusive evidence supporting this is still lacking [68]. A randomized clinical trial by Withayanukonkij et al. compared maxillary molar root resorption, intrusion amount, dentoskeletal changes, and maximum bite force between clear aligners and fixed appliances with mini-screws in 40 adults with anterior open bite [69]. After six months of treatment, significant root resorption was observed in both groups, with clear aligners causing 0.21-0.24 mm of resorption and fixed appliances causing 0.38-0.47 mm. Maxillary molar intrusion was 0.68 mm in the clear aligners group and 1.49 mm in the fixed appliances group, indicating that clear aligners caused significantly less root resorption and intrusion compared to fixed appliances. Additionally, clear aligners resulted in less overbite increase and bite closing but a greater increase in maximum bite force, with maximum bite force positively correlated with the amount of maxillary molar intrusion. In the same vein, Liu et al. analyzed the biomechanical effects of clear aligner therapy on root resorption and found that clear aligner therapy alone caused lingual tipping and extrusion of incisors, leading to concentrated stress on root surfaces and a predisposition to root resorption [70]. However, the addition of anterior mini-screws with elastics achieved incisor intrusion and palatal root torque, reducing the likelihood of root resorption by improving the biomechanical environment. Evidence suggests that while clear aligners can lead to root resorption, they cause significantly less root resorption compared to fixed orthodontic appliances.

An overview of the included studies that assessed the efficiency of clear aligners in managing anterior open bite cases, compared deep bite and anterior open bite treatments, or examined root resorption associated with overbite correction is depicted in Table 2.

**Table 2:** Summary of studies on clear aligners for anterior open bite, comparisons of deep and anterior open bite treatments, and root resorption from overbite correction.

Studies	Study design and sample	Study objectives	Main findings
Blundell et al. (2023) [59]	Retrospective study with 76 patients	Investigate the accuracy of Invisalign treatment in correcting anterior open bite	Clear aligners achieved approximately 66.2% of the programmed open bite closure compared to the ClinCheck (San Jose, CA: Align Technology) predictions. The study found that posterior occlusal bite blocks and prescribed movements did not significantly affect the efficacy of open bite closure. Two-week aligner changes resulted in a slight improvement in bite closure.
Moshiri et al. (2017) [60]	Retrospective study with 30 patients	Evaluate the vertical effects of non-extraction treatment of adult anterior open bite with clear aligners	Clear aligners effectively closed anterior open bites mainly through counterclockwise rotation of the mandibular plane, lower molar intrusion, and lower incisor extrusion.
Garnett et al. (2019) [61]	Retrospective study with 53 patients: 17 fixed appliances, 36 clear aligner	Compare fixed appliances and clear aligners in correcting anterior open bite and controlling the vertical dimension in adult hyperdivergent patients	No significant differences in the magnitude of overbite correction and changes in vertical dimension. Clear aligners showed slightly greater lower incisor extrusion. Both groups achieved open bite correction primarily through retroclination of upper and lower incisors.
Rask et al. (2021) [62]	Retrospective study with 44 patients for clear aligners and 22 patients for fixed appliance	Compare changes in vertical dimension and molar position in adult non-extraction anterior open bite with clear aligners and traditional fixed appliances	Traditional fixed appliances slightly extruded the lower molar and decreased overbite by 1.15 mm, while clear aligners showed slight mandibular backward rotation without significant changes in overbite. Both modalities resulted in increases in lower and total facial height.
Harris et al. (2020) [63]	Single-center retrospective study with 45 patients	Evaluate the dental and skeletal effects in the correction of anterior open bite with clear aligners	Open bite closure occurred due to a combination of maxillary and mandibular incisor extrusion and molar intrusion, with slight mandibular auto-rotation. Significant retraction of maxillary and mandibular incisors was also observed.
Suh et al. (2022) [64]	Retrospective study with 69 patients	Examine the effectiveness and mechanism of clear aligner therapy for correcting anterior open bite	Positive overbite was achieved in 94% of patients, with a mean change in overbite of 3.3 mm. Maxillary molar intrusion and mandibular incisor extrusion were key factors in open bite closure. The treatment mechanism varied by Angle’s classification, with Class II patients showing greater maxillary molar intrusion and Class III patients showing greater mandibular incisor extrusion.
Steele et al. (2022) [65]	Retrospective study with 53 patients	Compare the effectiveness of clear aligners and fixed appliances in treating anterior open bite	Clear aligners and fixed appliances were both effective in correcting anterior open bite. Fixed appliances showed greater improvement in molar intrusion, while clear aligners were more effective in incisor extrusion. The study emphasized the importance of proper case selection and treatment planning for optimal outcomes.
Talens-Cogollos et al. (2022) [66]	Retrospective descriptive-analytical study with 58 patients	Analyze and quantify molar intrusion after clear aligner treatment and its relationship with various variables	Approximately 74.2% of patients experienced molar intrusion with clear aligners. The intrusion was observed more in the mandibular molar (32.8%) than in the maxillary molar (15.5%). Significant relationships were found between molar intrusion and cephalometric variables like mandibular plane angle and facial axis.
Withayanukonkij et al. (2023) [69]	Randomized controlled trial with 40 patients	Compare changes in maxillary molar root resorption, intrusion amount, dentoskeletal measures, and maximum bite force between clear aligners and fixed appliances with mini-screw during molar intrusion	Maxillary molar intrusion and root resorption were significantly lower with clear aligners compared to fixed appliances with mini-screws. Clear aligners also showed less overbite and skeletal changes but increased maximum bite force more than fixed appliances.
Liu et al. (2021) [70]	Finite element analysis study	Analyze the biomechanical effects of clear aligner therapy on root resorption during anterior retraction, with and without mini-screws and elastics	Clear aligner therapy alone caused lingual tipping and extrusion of incisors, leading to concentrated stress on root surfaces, which could predispose to root resorption. The addition of mini-screws and elastics helped achieve incisor intrusion and palatal root torque, reducing the likelihood of root resorption.

Prospective developments

Future research in the management of deep bite and anterior open bite cases with clear aligner therapy should address several critical areas to enhance clinical outcomes and provide robust and generalizable evidence. Firstly, the potential for premature anterior occlusal contacts to limit mandibular autorotation should be carefully considered. In the Invisalign system, precise staging of incisor movements is crucial to avoid unwanted lingual tipping and incisor extrusion early in treatment. ClinCheck (San Jose, CA: Align Technology) approvals were not based on standardized protocol for movement sequencing among clinicians. This lack of standardization stresses the need for future studies to establish and evaluate standardized protocols for tooth movement sequencing. The debate on attachment design, tooth movement sequencing, and the extent of overengineering required in ClinCheck for specific clinical scenarios remains ongoing [30,71]. Open bite malocclusions are highly prone to relapse [72]. While the stability of correction of anterior open bite using mini-screws and fixed orthodontic appliances has been rigorously assessed, the long-term stability of Invisalign remains unclear [26]. Long-term follow-up studies for both treatment approaches are essential to ascertain their relative stability and inform recommendations for overcorrection and retention strategies required.

Future research should include high-quality randomized controlled trials that follow the CONSORT statement with rigorous methodology and appropriate sample sizes [73]. These trials should evaluate the treatment effectiveness of clear aligners in overbite correction compared to fixed appliances, incorporating different degrees of malocclusion and controlling for confounding variables. Additionally, it should use an objective assessment tool to evaluate treatment outcomes and post-treatment retention, measure periodontal health status before and after treatment, utilize cone-beam computed tomography to evaluate root resorption where appropriate, and employ validated tools to assess oral health-related quality of life. By addressing these areas, future research can provide more comprehensive insights into the effectiveness and long-term stability, safety, and biocompatibility of clear aligner therapy. This will ultimately guide clinicians in optimizing treatment plans for patients with deep bite and anterior open bite cases.

Limitations

Several limitations of the current review are acknowledged. Although the risk of potential bias associated with the included retrospective studies was reduced by applying rigorous inclusion and exclusion criteria to these studies, they are still prone to incomplete records and potential selection bias. Additionally, the sample for some studies was derived from a single clinician's practice, meaning the treatment modalities used may reflect the clinician's unique skills and expertise. This limits the generalizability of the results, and they should be interpreted with caution. The study also faced challenges due to heterogeneity in methodology, outcome reporting, and the types of aligners used. Most of the investigations focused solely on Invisalign, introducing potential bias due to the specific materials and planning software used. This may not be applicable to other clear aligner therapy manufacturers. Furthermore, the limitations of cephalometric analysis are well documented and may impact the findings. Differences in production process, material properties, and patient compliance with appliance wear can affect force levels and the predictability of tooth movements, further limiting the study's applicability [74]. Despite these limitations, this review offers valuable data for the management of patients with deep bite and anterior open bite malocclusion.

## Conclusions

The predictability of Invisalign in deep bite cases remains limited. Aligners face challenges in achieving accurate vertical movements, often requiring overcorrection and multiple refinements. Fixed orthodontic appliances remain superior for controlling and managing deep bite cases, suggesting that a hybrid approach or careful case selection might be essential for optimizing treatment outcomes with clear aligners. The closure of anterior open bite with clear aligner therapy is primarily achieved through significant incisor extrusion and lingual tipping, with some studies reporting non-clinically appreciable mandibular and/or maxillary molar intrusion. Despite the effectiveness of clear aligner therapy in anterior open bite correction, results show variability in the underlying mechanisms of bite closure. Overall, clear aligners offer a viable option for anterior open bite treatment, but the exact correction method remains debatable. The success of overbite correction is likely influenced by the clinician's experience with deep bite and anterior open bite cases, experience with clear aligner therapy, and the level of patient compliance.
